# Outbreak of Dengue Virus Serotype 3, Republic of the Marshall Islands, 2019–2021

**DOI:** 10.3201/eid3204.251135

**Published:** 2026-04

**Authors:** Tomás M. León, Jill McCready, Erlynda Chutaro, Janet McAllister, Bobson Solomon, Limb K. Hapairai

**Affiliations:** Pacific Island Health Officers’ Association, Honolulu, Hawaii, USA (T.M. León, L.K. Hapairai); Republic of the Marshall Islands Ministry of Health and Human Services, Majuro, Marshall Islands (J. McCready, E. Chutaro, B. Solomon); Centers for Disease Control and Prevention, Atlanta, Georgia, USA (J. McAllister); City of New Orleans Mosquito Termite & Rodent Control Board, New Orleans, Louisiana, USA (J. McAllister)

**Keywords:** dengue, DENV-3, viruses, vector-borne infections, zoonoses, mosquitoes, vector control, environmental sanitation, Marshall Islands, Pacific islands

## Abstract

During 2019–2021, the Republic of the Marshall Islands experienced a dengue outbreak involving 1,908 cases. Environmental sanitation helped stop transmission on Ebeye island, but transmission continued for >1 year on Majuro atoll. The Pacific region urgently needs to develop vector control capacity to address future dengue outbreaks.

Dengue, caused by dengue virus (DENV), is a vectorborne disease transmitted by *Aedes* spp. mosquitoes. DENV has 4 major serotypes (1–4); infection with one serotype protects against future infection with that serotype but leads to antibody-dependent enhancement and potentially more severe disease upon infection with a heterologous serotype (*1*). 

The primary global dengue vector is the *Ae. aegypti* mosquito, which has a widespread and ever-expanding range (*2*). In the Western Pacific, *Ae. albopictus*, *Ae. polynesiensis*, and *Ae. hensili* dengue vectors coexist with *Ae. aegypti* (*3*). Some Pacific Island countries and territories are dengue-endemic, but many others have periodic outbreaks in which DENV presumably is introduced by travelers returning from endemic areas (*4*). Introduction via infected mosquitoes brought by plane or boat also is possible and difficult to definitively rule out (*4*). In the Pacific, most DENV introductions are thought to be from returning residents rather than visiting tourists. 

Before 2019, the last major dengue outbreak in the Republic of the Marshall Islands (RMI) was during 2011–2012 and involved DENV-4 (*5*). The attack rate (AR) during that outbreak was 1.6% among laboratory-confirmed cases, and suspected AR was 3% (*5*). In that outbreak, 95% of tested samples (50% in children 0–4 years of age, 80% in children 5–9 years of age, >90% in persons >10 years of age) had evidence of secondary infection, showing a history of substantial DENV circulation in RMI (*5*). 

On May 15, 2019, a dengue case was identified in a patient on Ebeye island in the Kwajalein atoll of RMI. The case-patient had no travel history outside RMI. Another suspected case occurred on Ebeye on June 4, 2019. Subsequently, case counts rapidly increased and were detected across much of RMI, mainly on Kwajalein and Majuro atolls (Figure). Serotyping showed the outbreak was caused by DENV-3, which had not been recorded in RMI for 30 years, although DENV-3 possibly circulated in RMI during the mid-1990s or early 2000s (*6*). To investigate effectiveness of vector control strategies, we compared dengue ARs between the island of Ebeye, where systematic, islandwide vector control began soon after case detection, and the Majuro atoll, where human disease and vector surveillance is not routinely conducted. 

**Figure:**
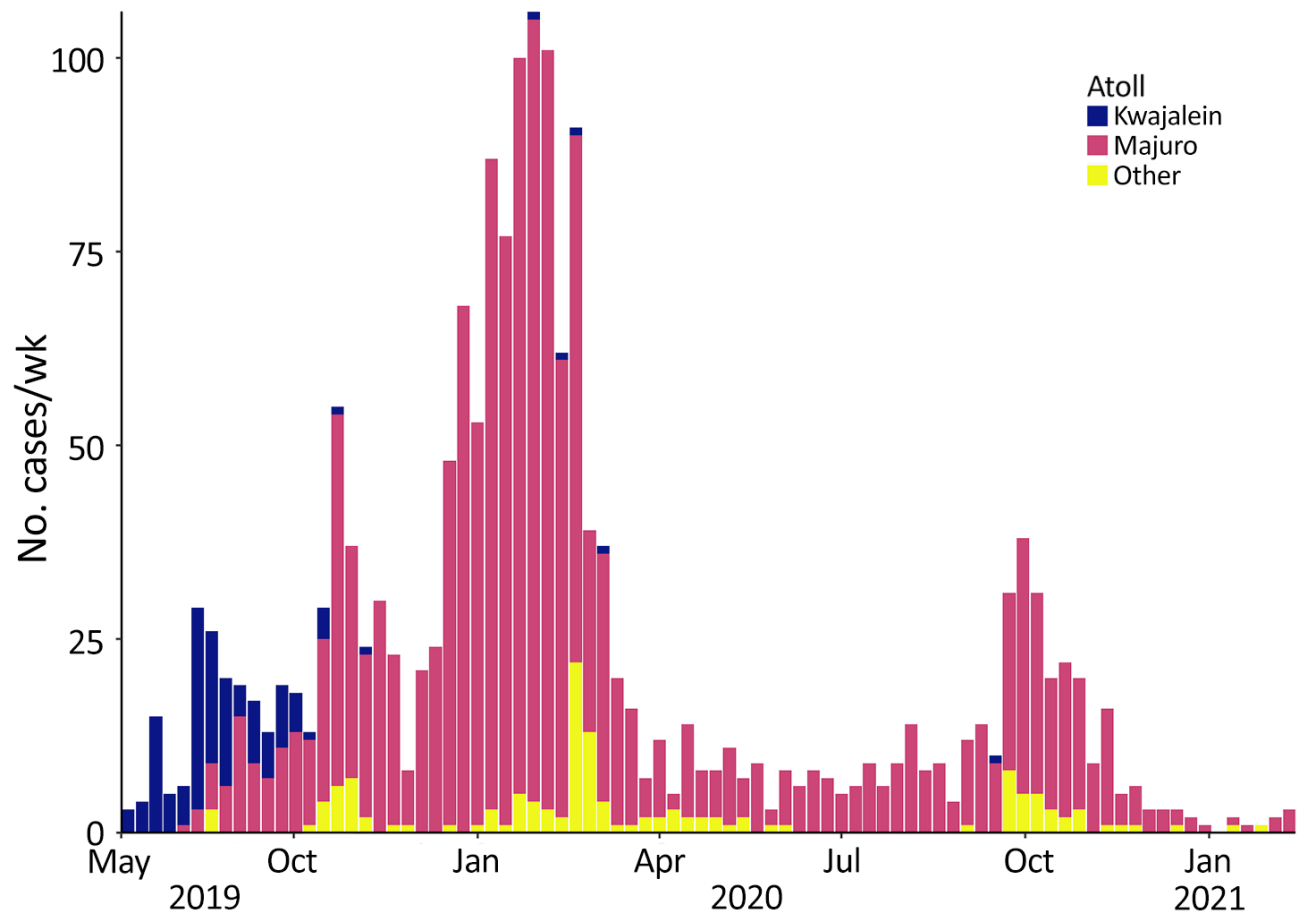
Number of confirmed cases per atoll in an outbreak of dengue virus serotype 3, Republic of the Marshall Islands, 2019–2021. Bars show weekly case counts. Although the first case was detected on Ebeye island in the Kwajalein atoll in May 2019, effective environmental sanitation and other vector control measures kept case counts low there, while cases spiked in Majuro atoll and persisted for >1 year.

## The Study

In response to dengue case detections, the Ministry of Health and Human Services (MOHHS) and the RMI Environmental Protection Authority (RMIEPA) initiated vector control activities across Ebeye on July 16, 2019. Ebeye government officials also began islandwide cleanup campaigns when dengue cases began increasing. RMIEPA used BG-Sentinel Mosquito Traps (Biogents, https://eu.biogents.com) for surveillance of adult mosquitoes and larvae or pupae to check for vectors before applying pesticides, when possible. *Ae. aegypti* mosquitoes were the only dengue vector found on Ebeye.

On Ebeye, RMIEPA applied indoor and outdoor residual adulticide bifenthrin or deltamethrin spray, and pyriproxyfen or *Bacillus thuringiensis* larvicide, on the basis of the larval survey, following the manufacturer’s labels. The vector assessment found *Ae. aegypti* mosquitoes in containers in about half of homes inspected (Table 1); the most productive larval mosquito containers were drums for storing water, mainly used for washing, not drinking. 

**Table 1:** Summary of immature mosquito vector assessment conducted on Ebeye island during outbreak of dengue virus serotype 3, Republic of the Marshall Islands, August 2019

Assessment	Value
Total no. households assessed	1,135
No. sampled for immature mosquitoes	105
House index*	50
No. containers inspected	476
Container index†	17
Breteau index‡	
Water cisterns	21
Drums	61
Buckets	6
Coolers	4
Abandoned appliances, boats, or vehicles	3
Bowls or pans	1
Overall	76

*Percentage of properties with >1 container positive for immature mosquitoes. †Percentage of containers positive for immature mosquitoes/100 properties. ‡Number of containers positive for immature mosquitoes/100 properties.

On August 6, 2019, the RMI government declared a state of emergency for the entire territory. MOHHS provided vector management efforts against *Aedes* spp. mosquito breeding sites through community engagement campaigns, including informational radio spots, social media posts, press reporting, and advocacy by traditional and ecclesial leaders. MOHHS and RMIEPA provided vector control to households with suspected and confirmed dengue cases.

PCR testing for RMI is only available in Hawaii, USA, as is common for many Pacific islands, so most (82%) confirmatory test results were ELISA of the nonstructural protein 1 antigen, 18% were DENV IgM, and <1% were PCR. Of note, most (59%) confirmed cases were among children 5–17 years of age (Table 2); however, only 44% of patients experiencing dengue-like illness during the outbreak were 5–17 years of age (Table 3).

**Table 2:** Characteristics of patients with confirmed dengue in an outbreak of DENV serotype 3, Republic of the Marshall Islands, 2019–21

Characteristics	No. (%) of cases, n = 1,908	Attack rate, %	No. (%) hospitalized
Sex			
F	882 (46)	4.3	313 (35)
M	1,018 (53)	4.7	401 (39)
Age group, y			
0–4	121 (6)	2.6	34 (28)
5–17	1,119 (59)	8.7	432 (39)
18–49	521 (27)	2.8	196 (38)
>50	104 (5)	1.7	28 (27)
Atoll			
Majuro	1,642 (86)	7.1	576 (35)
Kwajalein	132 (7)	1.3	88 (67)
Other	134 (7)	1.4	52 (39)
Disease severity			
Death	2 (0.1)	NA	NA
Hospitalized	716 (38)	NA	NA
Not hospitalized, recovered	1,190 (62)	NA	NA
Laboratory test results			
ELISA NS1 antigen–positive	1,565 (82)	NA	NA
ELISA IgM–positive	334 (18)	NA	NA
PCR-positive	9 (0.4)	NA	NA

*DENV, dengue virus; NA, not applicable; NS1, nonstructural protein 1.

**Table 3:** Characteristics of patients with dengue-like illness during outbreak of dengue virus serotype 3, Republic of the Marshall Islands, 2019–2021

Characteristics	No. (%) persons, n = 3,859	Attack rate, %	No. (%) hospitalized
Sex			
F	1,907 (49)	9.2	684 (36)
M	1,934 (50)	8.9	456 (24)
Age group, y			
0–4	412 (11)	8.7	50 (12)
5–17	1,685 (44)	13.1	454 (27)
18–49	1,381 (36)	7.3	290 (21)
>50	381 (10)	6.4	42 (11)
Atoll			
Majuro	3,403 (88)	14.7	636 (19)
Kwajalein	277 (7)	2.8	143 (52)
Other	179 (5)	1.9	57 (32)

Ebeye, where earliest dengue activity for this outbreak occurred, comprises ≈23% of the RMI population (*7*). By October 2019, case counts on Ebeye declined, and most dengue activity occurred on Majuro atoll, where ≈55% of the RMI population resides (*7*). Other atolls with documented cases were Ailinglaplap, Arno, Aur, Enewetak, Jaluit, Kili, Maloelap, Mili, Namu, Utrik, and Wotje. The overall dengue AR on Ebeye island was 1.3%, compared with the 7.1% AR on Majuro atoll, where vector control was only initiated in response to confirmed case identifications. After a spike in detected DENV transmission during December 2019–March 2020, dengue persisted on Majuro atoll with decreased case activity during March–September 2020 before cases increased in again in late 2020, preceding the eventual end of the outbreak in February 2021 (Figure).

RMIEPA conducted mosquito surveys on Majuro and other atolls, including Ailinglaplap, Arno, Aur, Enewetak, Jaluit, Kili, Kwajalein, Maloelap, Mili, and Wotje atolls, which confirmed *Ae. aegypti*, *Ae. albopictus*, and *Ae. marshallensis* mosquito vectors*.* Of note, those atolls had dengue ARs similar to Majuro. Although RMIEPA did not conduct mosquito surveys on Namu and Utrik atolls, the agency suspects those atolls likely had >1 of the *Aedes* spp. mosquito vectors.

The final confirmed case associated with this outbreak was recorded on February 20, 2021, resulting in a total of 1,908 laboratory-confirmed cases, among which 716 case-patients were hospitalized and 2 died. Officially, the dengue outbreak lasted 21 months, overlapping with the beginning of the COVID-19 global pandemic and associated travel restrictions that halted most international and intraisland travel beginning in March 2020. Of note, most dengue cases on Ebeye occurred in the first 2 months of the outbreak, suggesting successful vector control kept the overall AR low at 1.3%, compared with the 7.1% AR on Majuro atoll. Vector control was only initiated on Majuro in response to positive case identification, and DENV transmission persisted on the atoll. Recommendations by the US Centers for Disease Control and Prevention and the Pacific Island Health Officers Association for future dengue prevention and control efforts on the basis of the vector assessment included continued capacity building, environmental sanitation through improved waste management, chemical vector control, and improved domestic water collection and storage.

## Conclusions

RMI experienced an extended dengue outbreak during 2019–2021, overlapping with COVID-19 restrictions, suggesting that local transmission was sustained throughout the period unaided by additional case introductions. Initial cases were concentrated on Ebeye island in Kwajalein atoll, where vector control and environmental health activities helped mitigate local mosquito populations and curtail DENV transmission. Transmission persisted in Majuro atoll for an additional 18 months. Of note, Majuro is 4 times more densely populated than Kwajalein atoll (*7*), likely another contributing factor to the sustained transmission on Majuro compared with Kwajalein during this outbreak. The 2011–2012 dengue outbreak in RMI showed a similar AR between Kwajalein and Majuro (*5*). Compared with the 2011–2012 outbreak, confirmed dengue cases in the 2019–2021 outbreak had a younger age distribution and a higher concentration of cases in children 5–17 years of age. The relative lack of burden in the adult population suggests possible DENV-3 transmission in RMI in the 1990s, when that population might have experienced infection and therefore acquired immunity.

In summary, distribution of dengue cases across multiple atolls, several with high ARs, highlights the challenges for outbreak control in the Pacific and other regions where human disease and vector surveillance is not conducted regularly and systematically. Vector control and environmental health efforts on Ebeye successfully averted further DENV-3 transmission. Those findings support the need for building vector control capacity in the Pacific, where dengue outbreaks of this scale are likely to continue.
